# An integrative Bayesian network approach to highlight key drivers in systemic lupus erythematosus

**DOI:** 10.1186/s13075-020-02239-3

**Published:** 2020-06-23

**Authors:** Samaneh Maleknia, Zahra Salehi, Vahid Rezaei Tabar, Ali Sharifi-Zarchi, Kaveh Kavousi

**Affiliations:** 1 Laboratory of Complex Biological Systems and Bioinformatics (CBB), Department of Bioinformatics, Institute of Biochemistry and Biophysics (IBB), University of Tehran, Tehran, Iran; 2 Department of Immunology, School of Medicine, Tehran University of Medical Sciences, Tehran, Iran; 3 Department of Statistics, Allameh Tabataba’i University, Tehran, Iran; 4 School of Biological Sciences, Institute for Research in Fundamental Sciences, Tehran, Iran; 5 Department of Computer Engineering, Sharif University of Technology, Tehran, Iran; 6 Department of Stem Cells and Developmental Biology, Cell Science Research Center, Royan Institute for Stem Cell Biology and Technology, Tehran, Iran

**Keywords:** SLE, Signaling pathways, BNrich, Cross-platform normalization, Mixture model, Systems biology

## Abstract

**Background:**

A comprehensive intuition of the systemic lupus erythematosus (SLE), as a complex and multifactorial disease, is a biological challenge. Dealing with this challenge needs employing sophisticated bioinformatics algorithms to discover the unknown aspects. This study aimed to underscore key molecular characteristics of SLE pathogenesis, which may serve as effective targets for therapeutic intervention.

**Methods:**

In the present study, the human peripheral blood mononuclear cell (PBMC) microarray datasets (*n* = 6), generated by three platforms, which included SLE patients (*n* = 220) and healthy control samples (*n* = 135) were collected. Across each platform, we integrated the datasets by cross-platform normalization (CPN). Subsequently, through BNrich method, the structures of Bayesian networks (BNs) were extracted from KEGG-indexed SLE, TCR, and BCR signaling pathways; the values of the node (gene) and edge (intergenic relationships) parameters were estimated within each integrated datasets. Parameters with the FDR < 0.05 were considered significant. Finally, a mixture model was performed to decipher the signaling pathway alterations in the SLE patients compared to healthy controls.

**Results:**

In the SLE signaling pathway, we identified the dysregulation of several nodes involved in the (1) clearance mechanism (*SSB*, *MACROH2A2*, *TRIM21*, *H2AX*, and *C1Q* gene family), (2) autoantigen presentation by MHCII (*HLA* gene family, *CD80*, *IL10*, *TNF*, and *CD86*), and (3) end-organ damage (*FCGR1A*, *ELANE*, and *FCGR2A*). As a remarkable finding, we demonstrated significant perturbation in *CD80* and *CD86* to *CD28*, *CD40LG* to *CD40*, C1QA and C1R to C2, and C1S to C4A edges. Moreover, we not only replicated previous studies regarding alterations of subnetworks involved in TCR and BCR signaling pathways (*PI3K*/*AKT*, *MAPK*, VAV gene family, *AP-1* transcription factor) but also distinguished several significant edges between genes (PPP3 to NFATC gene families). Our findings unprecedentedly showed that different parameter values assign to the same node based on the pathway topology (the *PIK3CB* parameter values were 1.7 in TCR vs − 0.5 in BCR signaling pathway).

**Conclusions:**

Applying the BNrich as a hybridized network construction method, we highlight under-appreciated systemic alterations of SLE, TCR, and BCR signaling pathways in SLE. Consequently, having such a systems biology approach opens new insights into the context of multifactorial disorders.

## Introduction

Called as “the Great Imposter,” systemic lupus erythematosus (SLE) is a chronic and complex autoimmune disease with multisystem manifestations [1, 2]. The disease prevalence is estimated about 5 million people worldwide, and it occurs mostly before the age of 45 years [3–5]. The loss of central and peripheral tolerance to self, as a result of genetic susceptibility or environmental factors, is proposed to be a crucial first step in the SLE development [6, 7].

Certain lines of evidence suggested that a break in central B cell tolerance results in autoreactive clones to reach the periphery [8], which followed by autoantibodies’ production and immune complex deposition and finally damage the end organ [9, 10]. Likewise, an aberrant TCR signaling followed by hyper-responsiveness of T cells has been shown in SLE patients [11]. The genome-wide association studies (GWAS) coupled with gene expression profiling data shed light on the critical role of genes involved in B cell receptor (BCR) and T cell receptor (TCR) signaling pathways in SLE pathogenesis [7]. Despite numerous strong-minded studies have been done, the SLE pathogenesis is still far from clear. Many studies used bioinformatics approach such as enrichment analysis and determined pathways implicated in multifactorial disease pathogenesis. To the best of our knowledge, there is no study with deep insight into the implicated pathways in SLE.

Towards a better intuition of molecular mechanisms involved in multifactorial immune-related diseases especially SLE, lots of researchers acknowledge the gene network approach which helps to prioritize main driver genes and pathways, to propose a better drug development [12]. Bayesian networks (BNs), as a worthwhile powerful gene network construction methods, integrate and model biological data with causal relationships [13–17]. By applying gene expression data of SLE patients, Li et al. recently reconstructed a BN, in which prior edges were randomly sampled based on the text-mining in SLE pathogenesis [12]. Remarkably, some methods such as BNrich were also developed based on BN properties [18] which identifies significant genes (nodes) and biological relationships (edges). The BNrich method has two key concepts: (1) the structure of BN is reconstructed by the signaling pathway structure, and (2) the expression level of the gene (node) is modeled as a regression function of expression levels of its upstream genes (parents) [18].

As a method that is widely used to integrate gene expression data, *cross-platform normalization* (CPN) (1) increases sample sizes and improves gene signature selection [19–23], (2) rises the heterogeneity of the overall estimate, and (3) decreases the effects of individual study-specific biases [22, 23]. On the other hand, the mixture model, known as a model averaging method, is well documented to integrate node and edge parameters based on their distribution [24].

With systems biology approach, we aimed to illustrate the superiority of using CPN and mixture model method and the BNrich to better understand new aspects of underlying molecular mechanisms in the pathogenesis of complex diseases such as SLE. Here, we concentrated on SLE, BCR, and TCR signaling pathways, which are among the most enriched pathways in SLE, to highlight significant alterations of those pathways in SLE patients compared to healthy controls. Besides the altered gene expression level, we demonstrated several significant intergenic relationships which can be proposed as effective targets for therapeutic intervention in SLE patients.

## Methods

**Fig. 1:**
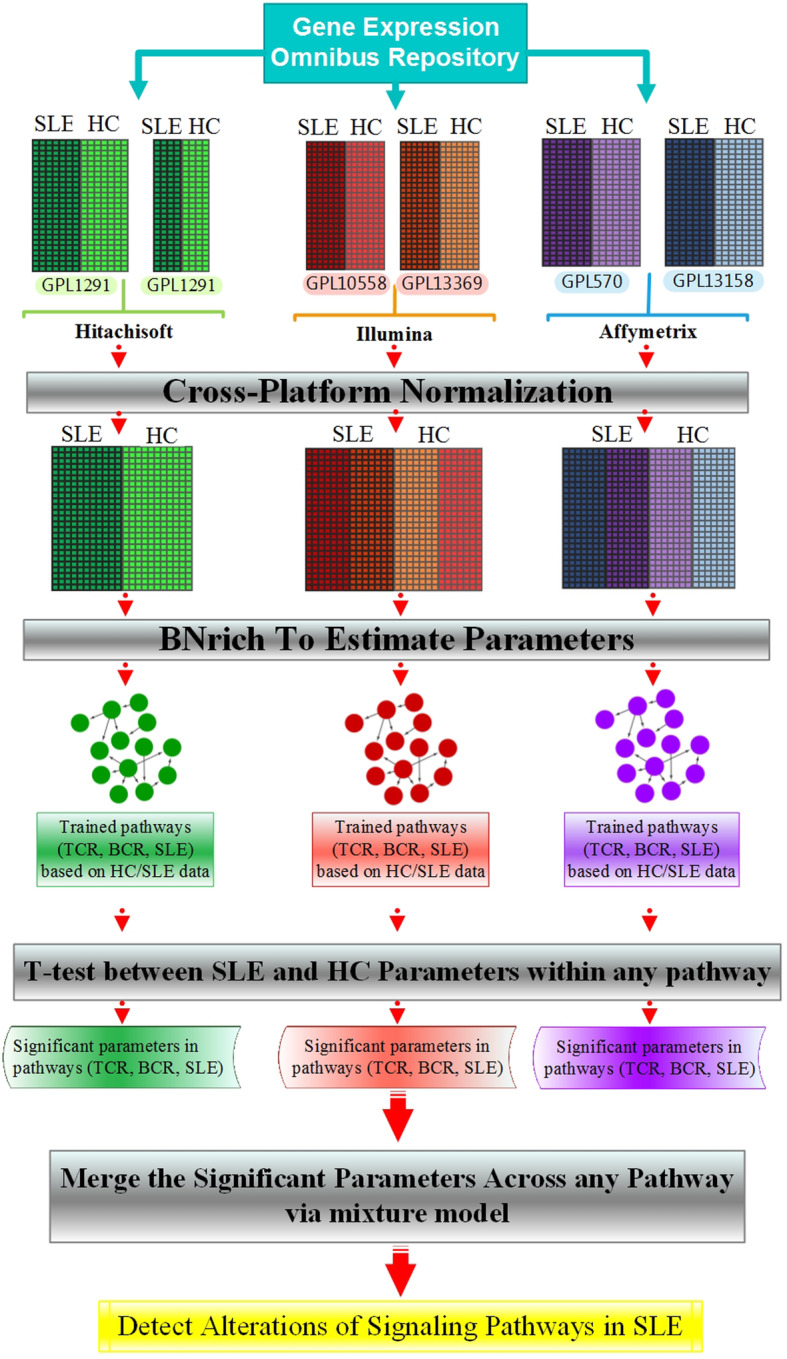
Workflow and analysis procedure to identify signaling pathway alterations in SLE patients. At the first step, human peripheral blood mononuclear cell (PBMC) microarray datasets associated with SLE, which were generated using three platforms, were downloaded and each paired data related to the same platform integrated by CPN method. Subsequently, SLE, TCR, and BCR signaling pathways were employed as structures of BNs and trained by three major datasets independently by BNrich method. Afterwards, the differences between any paired corresponding parameters related to patients and controls were examined by independent *t* test. Consequently, the significant parameters merged by the mixture model to achieve the key driver parameters in studied pathways

The human peripheral blood mononuclear cell (PBMC) microarray datasets (*n* = 6) associated with SLE were downloaded and integrated by CPN method. Subsequently, we employed SLE, TCR, and BCR signaling pathways as structures of BNs and we determined significant node and edge parameters by BNrich method in each dataset independently. Consequently, significant parameters of all datasets were merged through mixture model method and key driver parameters in the selected pathways were defined (Fig. 1).

### Gene expression datasets

The human PBMC microarray datasets that contain both SLE patient and healthy control (HCs) samples, published or updated in 2010–2019, were downloaded from the Gene Expression Omnibus (GEO) database: GSE 17755 [25], GSE 12374 [26], GSE 50772 [27], GSE 81622 [28], GSE 121239 [29–31], and GSE 126307 [32]. We described the details of the data in Table 1.

**Table 1:** Description of the datasets used in the study

No.	GSE no.	GPL/platform	No. of sample		Cell type	Update (year)	Race
			SLE patients	Controls			
1	17755	1291/Hitachisoft	22	55	PBMC	2010	Japanese
2	12374	1291/Hitachisoft	11	6	PBMC	2012	Japanese
3	50772	570/Affymetrix	61	20	PBMC	2015	Unknown^a^
4	81622	10558/Illumina	30	25	PBMC	2016	Unknown^b^
5	121239	13158/Affymetrix	65^c^	20	PBMC	2018	Caucasian/African American^d^
6	126307	13369/Illumina	31	9	PBMC	2019	Several races^e^

^a^The data were collected in the USA/South San Francisco, but the race of subjects is unknown

^b^The data were collected in the USA/Dallas, but the race of subjects is unknown

^c^Only the data related to the first visit (v1) samples of any patient were entered in the analysis

^d^This subset of the samples is derived from a preliminary dataset which were collected in the USA/Johns Hopkins University School of Medicine Institutional, and most of the race of subjects (92.8%) are Caucasian and African American

^e^The data collected in USA/Dallas, but the race of subjects is Australian, Australian (Irish/Scottish descent), born in India—ethnicity unknown, Caucasian, Caucasian/Japanese, English, Filipino, Indian, Iraqi, Latin American, Persian, Spanish, and White Australian/Anglo-Celtic

### Cross-platform normalization

Each expression dataset was preprocessed and normalized by *normalizeQuantiles* function from R package *limma* [33]. Then, we performed CPN [23] with *Reduce* function in R to integrate each paired gene expression data emanated from the same platform. Finally, we have three major datasets from Affymetrix, Illumina, and Hitachisoft; each platform has a SLE patient group and a HC group. Afterwards, the empirical Bayes method (ComBat) from the R package *sva* was used for batch effect removal [34].

### BNrich approach

To reconstruct BN structures, the SLE (hsa:05322), TCR (hsa:04660), and BCR (hsa:04662) signaling pathways were implemented. All of the pathways were extracted directly from the KEGG database (Release 90.0, April 1, 2019) [35].

In the parameter estimate step, the mean value of the expression for each gene (node) can be modeled as a linear regression of its parents’ (upstream) gene expression [18]. When *Y* gene has *X*_1_, *X*_2_…*X*_*p* − 1_ parents in the network, and ${\hat{Y}}_s$ and ${\hat{Y}}_h$ describe the estimations of *Y* for the SLE patient and HC datasets, respectively, they can be modeled as follows:

1 $$ {\displaystyle \begin{array}{c}\ {\hat{Y}}_s={\beta}_{s0}+{\beta}_{s1}{\mathrm{X}}_1+{\beta}_{s2}{\mathrm{X}}_2+\dots +{\beta}_{s\left(p-1\right)}{\mathrm{X}}_{p-1}+{\varepsilon}_c\\ {}{\hat{Y}}_h={\beta}_{h0}+{\beta}_{h1}{\mathrm{X}}_1+{\beta}_{h2}{\mathrm{X}}_2+\dots +{\beta}_{h\left(p-1\right)}{\mathrm{X}}_{p-1}+{\varepsilon}_h\end{array}} $$

In the SLE- and HC-associated trained BNs, the constant coefficient *β*_(s/h)0_ demonstrates the expression level of *Y* gene, known as a node parameter. The coefficients of *X*_1_, *X*_2_…*X*_*p* − 1_ are the parameters of the edge in the trained BNs which reveal a biological relationship between two genes, *Y* and *X*. For example, the value of *β*_*s*3_ is the edge parameter between *Y* and *X*_3_ genes in the network structure related to SLE patient data and shows the value of the biological relationship between these two genes. The *ε*_*c*_ and *ε*_*h*_ are the residual values.

Using Eq. 1, the node and edge parameters of the BN structures (derived from SLE, BCR, and TCR signaling pathways) were estimated for SLE patient- and HC-gene expression datasets of the three related platforms, separately. Subsequently, we compared the parameters of trained networks in SLE patients (*β*_*s*_) with HCs (*β*_*h*_) by independent *t* test [36] and gained the *β*^∗(*j*)^ (*j* ∈ {*A*, *I*, *H*}) which is the significant parameter based on false discovery rate (*FDR*_*j*_ < 0.05) for Affymetrix (A), Illumina (I), and Hitachisoft (H) datasets.

The estimated parameters of BNs have the following distribution [37]:

2 $$ \beta \approx N\left(\hat{\beta},{\sigma}^2{\left({X}^TX\right)}^{-1}\right) $$

Accordingly, the significant parameters had been distributed as follows:

3 $$ {\beta}_s-{\upbeta}_h={\beta}^{\ast };{\beta}^{\ast (j)}\approx N\left(\mu \left({\beta}_s\right)-\mu \left({\beta}_h\right),\operatorname{var}\left({\beta}_s\right)+\operatorname{var}\left({\beta}_h\right)\right)\ j\in \left\{A,I,H\right\} $$

Therefore, we had up to three significant parameters for each node (or edge) which are *β*^∗(*A*)^, *β*^∗(*I*)^, and *β*^∗(*H*)^.

### Merge significant parameters via mixture model

By using the mixture model approach [38], we merged the significant parameters of three platform-associated datasets and calculated the significant final parameters, both at the node and edge level. The final parameters, *β*_*f*_, have a normal distribution, and their mean and variance were defined by:

4 $$ {\displaystyle \begin{array}{c}\mu \left({\beta}_f\right)=\sum \limits_{j\in \left\{A,I,H\right\}}{p}_j\left(\mu \left({\beta}^{\ast (j)}\right)\right)\\ {}\mathrm{Var}\left({\beta}_f\right)={\sum}_{j\in \left\{A,I,H\right\}}{p}_j\left(\operatorname{var}\left({\beta}^{\ast (j)}\right)+{\mu}^2\left({\beta}^{\ast (j)}\right)\right)-{\mu}^2\left({\beta}_f\right)\\ {}{p}_j=\frac{N_j}{N_A+{N}_I+{N}_H}\kern1em j\in \left\{A,I,H\right\}\end{array}} $$

where *N*_*A*_ = 166, *N*_*I*_ = 95, and *N*_*H*_ = 94 were the number of subjects in Affymetrix, Illumina, and Hitachisoft. Since the range of the variances across final parameters is varied, their mean is divided by the standard deviation to achieve comparable parameters. Hence, we plotted the figures based on the following definition:

5 $$ {\mu}^{\ast}\left({\beta}_f\right)\triangleq \frac{\mu \left({\beta}_f\right)}{\sqrt{\mathrm{Var}\left({\beta}_f\right)}} $$

If the parameter *β*^∗(j)^ in each dataset was not significant, its mean and variance were considered to be zero in calculating the corresponding final parameter. When the *β*_*f*_ was associated with the node parameters, the sign of *μ*^∗^(*β*_*f*_) indicated upregulation (+) or downregulation (−) of the gene and the absolute value of *μ*^∗^(*β*_*f*_) was used to measure the value of activation or inhibition. Where the *β*_*f*_ was associated with the edge parameters, the sign of *μ*^∗^(*β*_*f*_) indicated increasing (+) or decreasing (−) biological function and the absolute value of *μ*^∗^(*β*_*f*_) was used to measure the value of these increased or decreased functions.

## Results

### Data integration using CPN

**Fig. 2:**
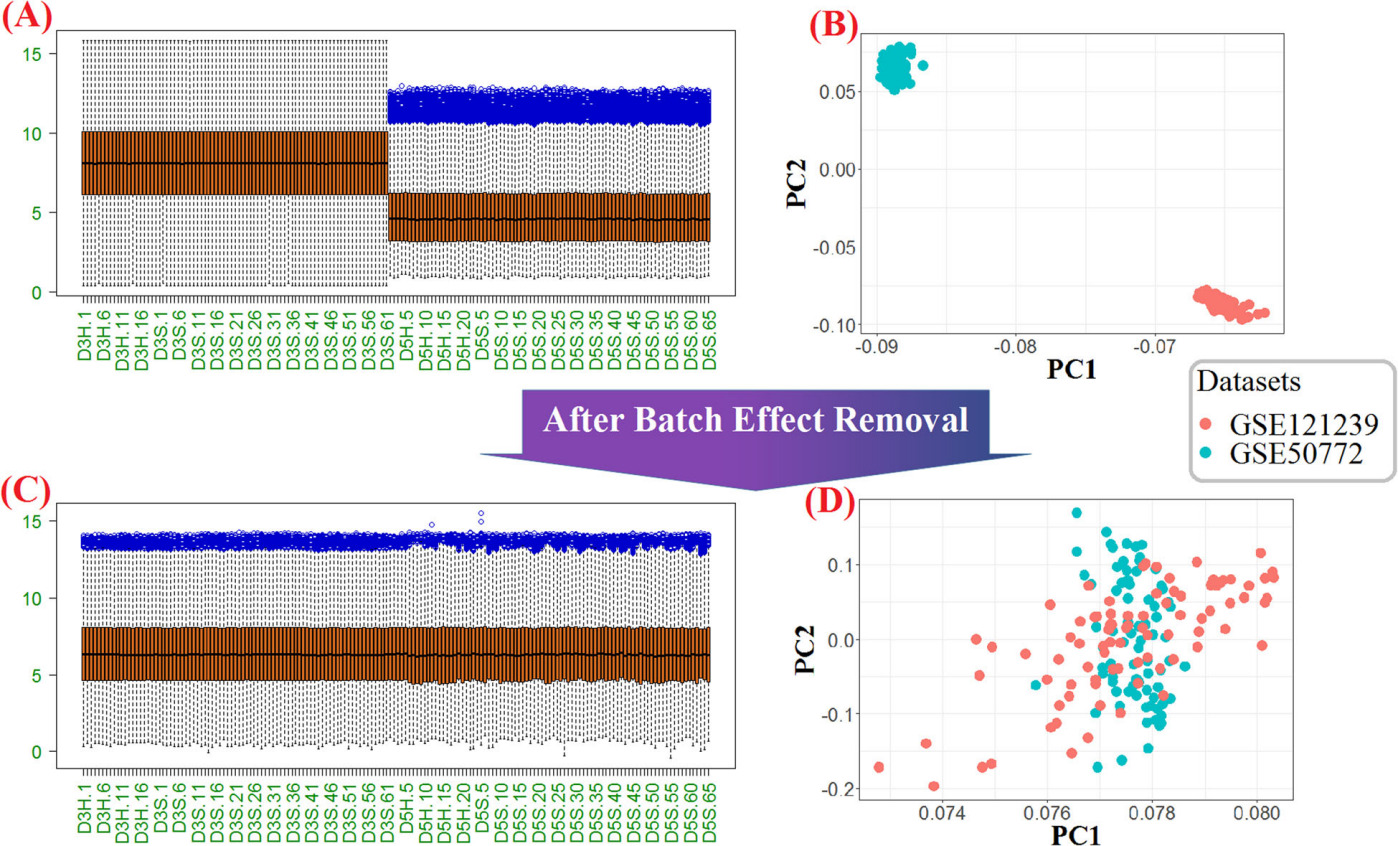
Graphical demonstration of the batch effect removal using ComBat for Affymetrix platforms. Boxplot (**a**) and PCA plot (**b**) show the gene expression distributions and the samples of microarray datasets before batch effects removal respectively. As the same way, Boxplot (**c**) and PCA plot (**b**) show the corresponding concepts after batch removal. The boxplots distributions show the normalization and decreasing technical diversities between datasets; and in the horizontal axis, the j^th^ healthy control subjects and the j^th^ SLE patient in the i^th^ dataset were illustrated with DiH.j and DiS.j, correspondingly. In the PCA plots, each dot represents one sample, and the color indicates its dataset

After downloading the six mentioned datasets (Table 1), to reduce the batch effect generated between different arrays, each paired data emanated from the same platform was integrated using the ComBat algorithm. The efficiency of the ComBat process in our integration for batch removal can be verified by the comparative boxplots and principle component analysis (PCA) plots (Fig. 2 and Additional file 1).

We did not observe any genes lost after performing CPN on Illumina and Hitachisoft related data, while in the Affymetrix platform, information of about 5.4% and 8.5% of genes in the datasets GSE 50772 and GSE 121239 were lost correspondingly. Considering the CPN benefits, we pursue of using all 166 samples of Affymetrix platform simultaneously instead of analyzing 81 or 85 individual samples (Table 2).

**Table 2:** The percentage of lost genes after running CPN

GSE no.	Platform	Genes (***n***)	Genes after CPN (***n***)	Lost genes (%)
50772	Affymetrix	19,689	18,627	5.4
121239		20,351		8.5
81622	Illumina	30,500	30,500	0
126307		30,500		0
17755	Hitachisoft	13,102	13,102	0
12374		13,102		0

### Identify significant parameters using BNrich

To identify significant parameters, including nodes and edges, in SLE patients against HCs in every major dataset, the SLE, TCR, and BCR signaling pathways were utilized as structures of BN and significant parameters (nodes and edges) were determined using BNrich method. Table 3 presents the number of significant node and edge parameters in the three dataset-associated platforms based on FDR < 0.05 for SLE, TCR, and BCR signaling pathways.

**Table 3:** The number of all significant node and edge parameters of BNs extracted from SLE, TCR, and BCR signaling pathways obtained via BNrich

Signaling pathways	FDR < 0.05					
	Affymetrix		Illumina		Hitachisoft	
	Nodes	Edges	Nodes	Edges	Nodes	Edges
**SLE**	73	4	108	11	50	7
**TCR**	82	161	81	120	75	75
**BCR**	57	103	68	91	56	60

According to our results, 34 significant nodes and the C1QB → C2 edge were common among all datasets in the SLE pathway. Across datasets of all platforms, 53 nodes and 26 edges of the TCR signaling pathway and 36 nodes and 30 edges of the BCR signaling pathway were common. All significant parameters of different platforms, based on three examined signaling pathways, are presented in Additional file 2.

### Determine the key driver parameters of signaling pathways via mixture model

To distinguish the altered signaling pathway parameters in SLE patients compared to HCs, the identified significant parameters of all dataset merged by the mixture model method (Eqs. 4 and 5), the range of *μ*^∗^(*β*_*f*_) in all nodes and edges are represented in Table 4.

**Table 4:** The minimum and maximum final parameter values of nodes and edges in three signaling pathways

		SLE		TCR	BCR		
		Nodes	Edges	Nodes	Edges	Nodes	Edges
*μ*^∗^(*β*_*f*_)	**Min**	− 1.70	− 0.46	− 1.68	− 1.01	− 1.39	− 0.96
	**Max**	2.37	0.55	1.43	1.12	1.43	0.94

The three nodes and edges that have the extreme values of *μ*^∗^(*β*_*f*_) in each desired pathway are illustrated in Table 5, and the all *μ*^∗^(*β*_*f*_) represented in Additional file 2.

**Table 5:** The top three nodes and edges of the studied pathways

	Nodes		Edges	
	Down	Up	Increasing biological function	Decreasing biological function
**SLE**	SNRPD3	FCGR1A	C1R → C4A	C1QA → C2
	HLA-DPB1	CTSG	C1QB → C4A	CD86 → CD28
	HLA-DMA	ELANE	C4B → C3	C1S → C4A
**TCR**	CD3E	BCL10	RASGRP1 → NRAS	NCK2 → PAK3
	PPP3CC	PAK5	CBLB→FYN	MAP2K1 → MAPK1
	CBLB	MAPK14	PPP3CB → NFATC3	CD3E → FYN
**BCR**	INPP5D	BCL10	AKT3 → IKBKG	SYK → PIK3AP1
	PPP3CC	PIK3AP1	MAPK1 → FOS	MAP2K1 → MAPK1
	CD81	IFITM1	PPP3CC → NFATC1	VAV1 → RAC2

Among genes involved in the clearance mechanism of SLE pathway, *TRIM21*, *H2AX*, *C1QA*, *C1QB*, *H2BC12*, and *H2BC12* were mostly upregulated. However, *SNRPD3*, *SSB*, *MACROH2A2*, *GRIN2B*, and *C4B* were mostly downregulated. In antigen presentation process mediated by MHCII, nodes such as *HLA*-*DPB1*, *HLA*-*DMA*, *CD80*, *IL10*, and *TNF* and three edges, *CD80* and *CD86* to *CD28* and *DD40LG* to *CD40*, were found to be dysregulated.

**Fig. 3:**
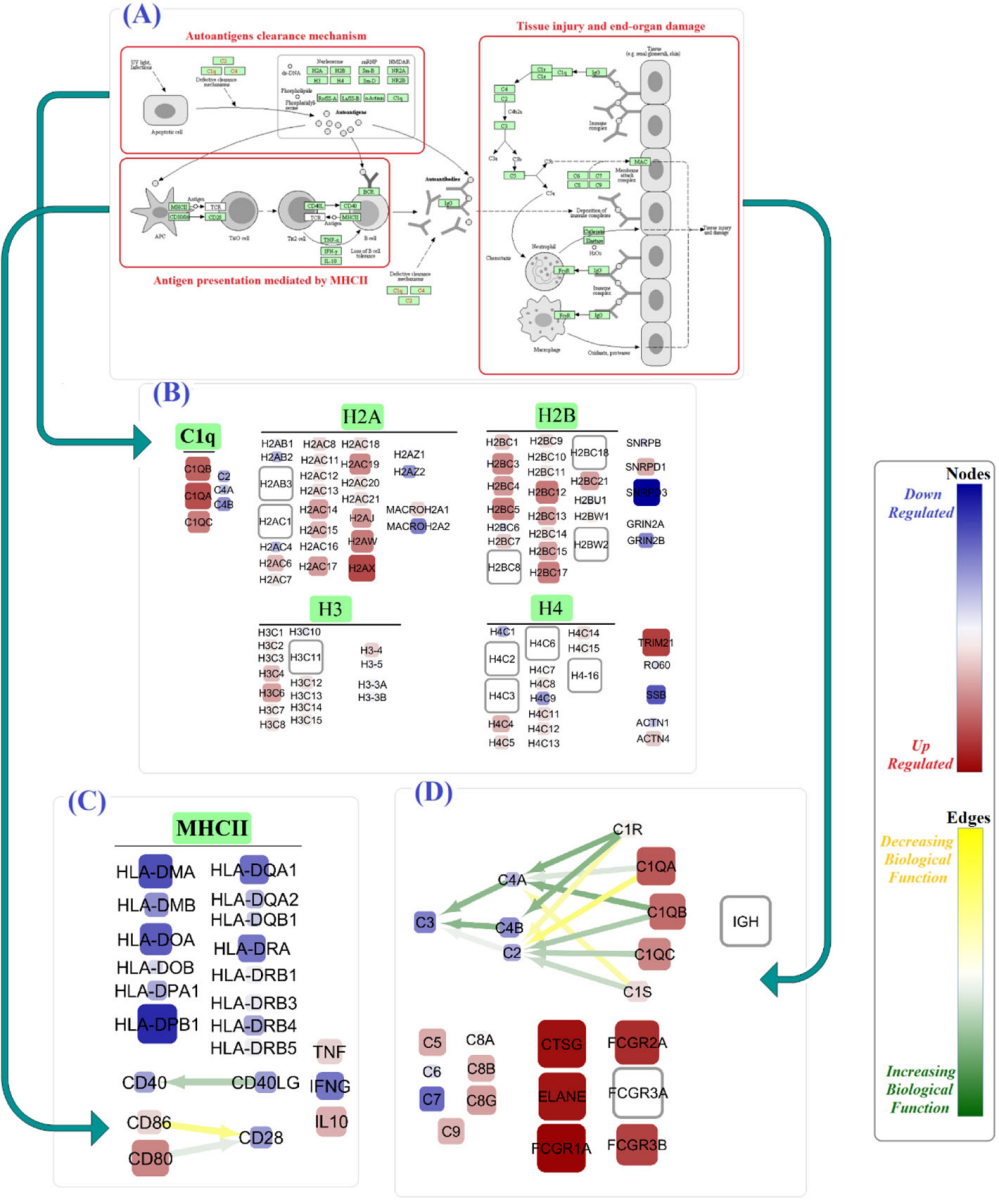
The alterations in SLE signaling pathway based on calculated final parameters. The representation design of KEGG-extracted SLE signaling pathways (**a**), genes involved in autoantigen clearance (**b**), antigen presentation mediated by MHCII (**c**), and tissue injury and end-organ damage (**d**). The color and size of nodes reflect the values and the absolute values of the *μ*^∗^(*β*_*f*_), respectively, and it is equivalent to the upregulation or downregulation of genes. Moreover, the color of the edges reflects the values of the *μ*^∗^(*β*_*f*_), respectively, and it is equivalent to the increasing or decreasing biological function

In addition, our analysis showed that among genes which participated into damaging of the end organ, the expression of genes like *FCGR1A*, *CTSG*, *ELANE*, *FCGR3B*, *FCGR3B*, and *C1QA* were increased and the expression of *C7*, *C3*, *C4B*, *C2*, and *C4A* genes were decreased. Particularly, in that functional part of SLE pathway, we also found dysregulation of several important edges like *C1R*, *C1S*, and *C1QB* to *C2* (Fig. 3 and Additional file 2).

**Fig. 4:**
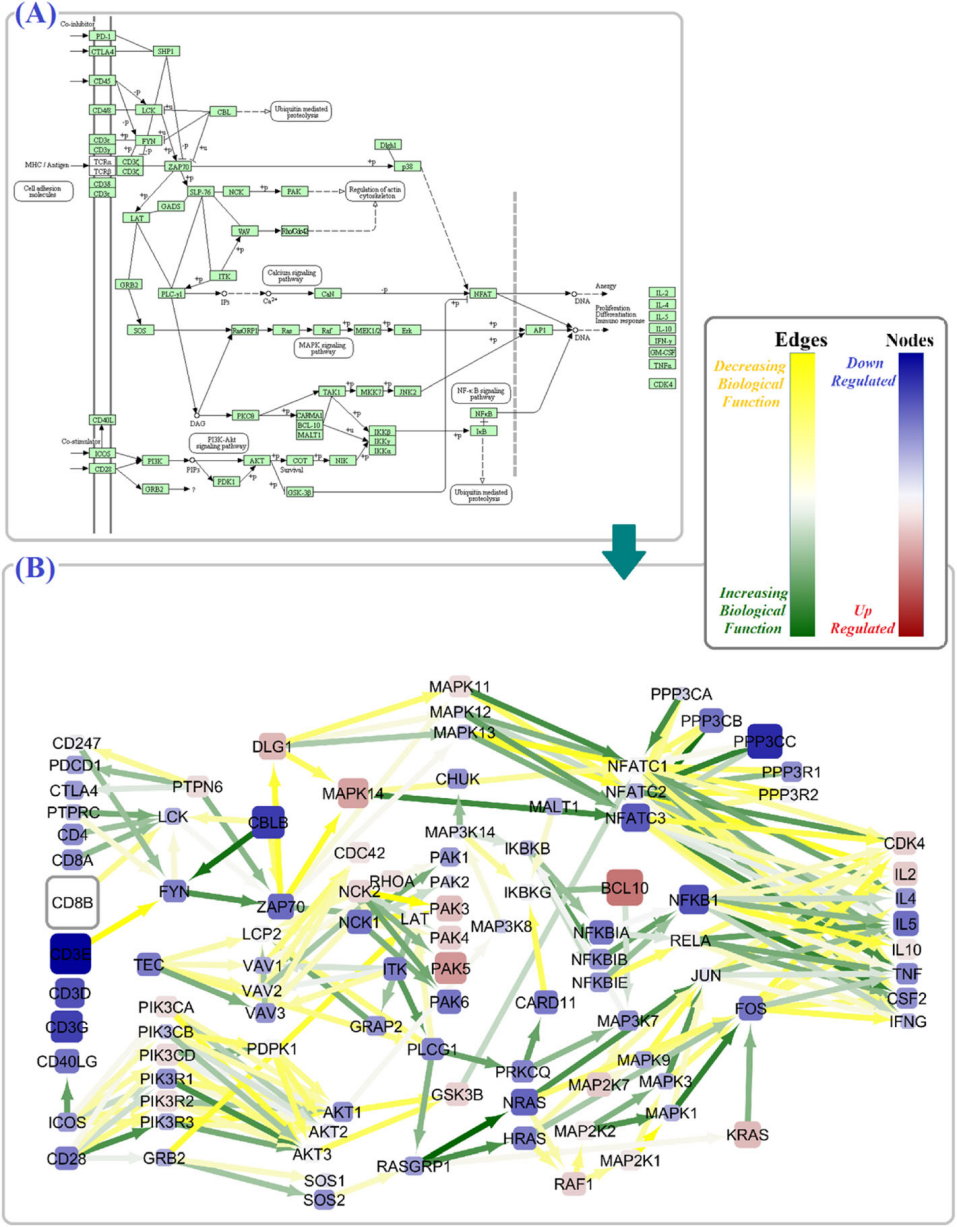
The alterations in TCR signaling pathway. The schematic figure of the TCR signaling pathway in KEGG (**a**), and the TCR signaling pathway alterations based on calculated final criteria (**b**). The color and size of nodes reflect the values and the absolute values of the *μ*^∗^(*β*_*f*_), respectively, and it is equivalent to the upregulation or downregulation of genes. Moreover, the color of the edges reflects the values of the *μ*^∗^(*β*_*f*_), respectively, and it is equivalent to the increasing or decreasing biological function

**Fig. 5:**
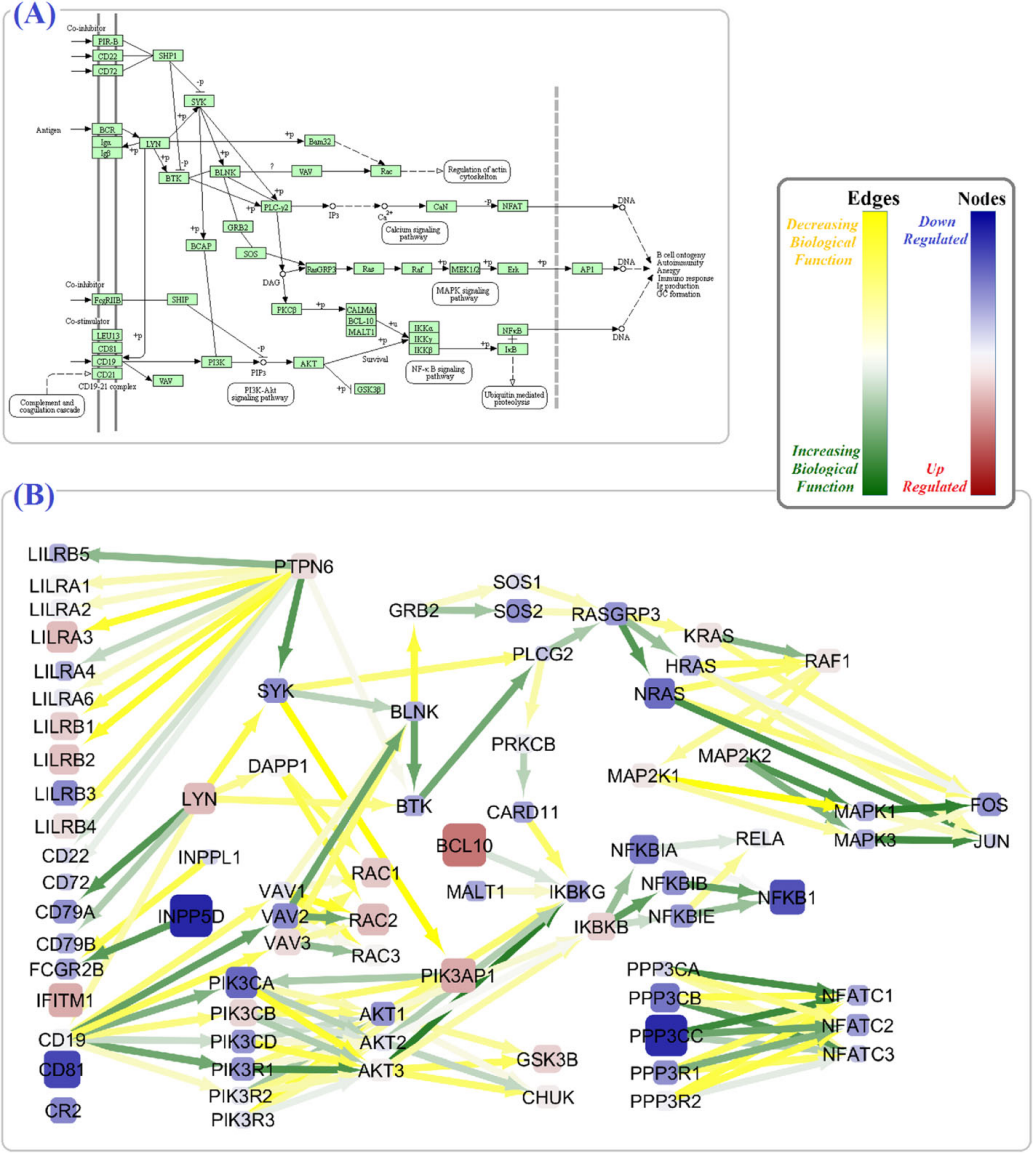
The alterations in the BCR signaling pathway. The schematic figure of the BCR signaling pathway in KEGG (**a**), and the BCR signaling pathway alterations based on calculated final criteria (**b**). The color and size of nodes reflect the values and the absolute values of the *μ*^∗^(*β*_*f*_), respectively, and it is equivalent to the upregulation or downregulation of genes. Moreover, the color of the edges reflects the values of the *μ*^∗^(*β*_*f*_), respectively, and it is equivalent to the increasing or decreasing biological function

By integrating BCR and TCR signaling pathway information with gene expression data, we identified many of the altered key driver genes in these pathways among SLE patients. Our results revealed 46 shared dysregulated nodes (e.g., *AKT1*, *AKT2*, *AKT3*, *MALT1*, *MAPK3*, SOS) and 64 common dysregulated edges (e.g., *PIK3CA* to *AKT1* and *AKT2*, *IKBKB* to *NFKBIA*, *NFKBIB* and *NFKBIE*) between these two pathways (Figs. 4 and 5 and Additional file 2).

## Discussion

In the context of identifying SLE gene signature, previous studies have been limited to gene expression level regardless of intergenic relationship. In the present study, for the first time, through data aggregation via BNrich, CPN, and mixture model method, we merged several diverse gene expression data into SLE, TCR ,and BCR signaling pathways and unraveled key genes and intergenic relationships that may serve as effective targets for therapeutic intervention in SLE patients. By using the CPN method, we increased the sample size and decreased the heterogeneity of the samples in comparison to previous studies [25–32]. According to BNrich properties, we could integrate two levels of biological data, gene expression and signaling pathways. Hence, the value of the node and edge parameters in addition to being influenced by gene expression data also depends on the topology of the underlying pathway [18]. For instance, in the Illumina platform, the mean value of the final parameter in *PIK3CB* gene was 1.7 and − 0.5, and the mean value of the final parameter in edge PPP3CC → NFATC3 was − 0.07 and 0.12, in TCR and BCR signaling pathways, correspondingly (Additional file 2). Moreover, we used the mixture model, as a model averaging method, to determine the key driver parameters across all datasets. In contrast to model selection, the mixture model dramatically minimizes the risk relative to selection [24].

First, we investigated SLE signaling pathway (hsa:05322), which included three sections: (1) autoantigen clearance mechanism, (2) antigen presentation mediated by MHCII, and (3) tissue injury and end-organ damage. The uncleaned apoptotic components which recognized by both the innate and the adaptive immune systems can trigger the pathogenesis of SLE. Therefore, the dysregulation of effective genes involved in the clearance of apoptotic components has a fundamental role in SLE [39–43]. In the same vein, we showed significant upregulation of 69 genes which include *TRIM21*, *C1Q* gene family, and *SNRPD1* and significant downregulation of 15 genes which include *SNRDP3*, *SSB*, and *GRIN2B* (Fig. 3b). Over the recent years , *TRIM21*, *SSB*, and *C1Q* have gained particular attention in SLE [44–46]. In line with earlier studies, we also underscored the dysregulation of histone gene family (H2A, H2B, H3, and H4) [47]. The clearance procedure followed by antigen presentation was mediated by MHCII. While *CD80*, *CD86*, *IL10*, and *TNF-α* as key mediators of this pathway were distinctively activated, the HLA gene family members (*DPB1*, *DQA1*, *DMA*, etc.) showed the significant reduction (Fig. 3c). The association of HLA genes with childhood- and adult-onset SLE was represented recently [48]. Besides, the elevated level of serum *TNF-α* has been shown in SLE patients. Using anti-TNF monoclonal antibodies like infliximab and etanercept to treat SLE patients further confirmed the importance of TNF as a targeting agent [49]. Despite the negative results for *CD40LG* and *CD40* node parameters, the edge parameter between them showed a positive biological function. Therefore, we further highlight the therapeutic effect of *CD40-CD40LG* pathway blockade in SLE [50]. Although *CD28* expression was reduced, *CD80* → *CD28* biological function was enhanced significantly (Fig. 3c). At the end of the SLE signaling pathway, end-organ damage occurs via dysregulation of several genes. Among them, the deficiencies of early complement proteins, including *C1*, *C4*, and *C2*, were strongly associated with the development of SLE [51]. Our results showed that not only the parameters of *C3*, *C7*, and *C4B* were reduced but also *C1R* → *C2*, *C1QA* → *C2*, and *C1S* → *C4A* biological functions were lessened (Fig. 3d). Additionally, our findings showed augmented *ELANE*, *FCGR1A*, *FCGR2A*, *FCGR3B*, and *CTSG* node parameters. By applying a multicohort analysis of 7471 transcriptomic profiles, Haynes et al. have just introduced *ELANE* as an “Under-appreciated SLE MetaSignature.” [52] In a recent study, the *FCGR2A* polymorphisms have been linked to SLE susceptibility in Mexican patients [46].

As outlined earlier, the fundamental importance of BCR and TCR signaling pathways in SLE pathogenesis has been well established via both clinical and experimental evidence [53–55]. In the TCR signaling pathway, we observed lower expression of several genes like *CBLB*, *PPP3CC*, *NFKB1*, *CD3E*, and *ZAP70*. Recently, a study by Matsuo et al. revealed that a genetic mutation in *ZAP70* resulted in the TCR signaling defect which followed by T follicular helper (Tfh) cell development and the manifestation of lupus-like systemic autoimmunity [56]. Having several significant edges with the other nodes proposes *ZAP70* as a hub gene of the TCR signaling pathway implicated in SLE pathogenesis. Interestingly, the perturbation in TCR culminated to enhanced expression of *CDK4*, *IL2*, and *IL10* genes. In the BCR signaling pathway, the most up- and downregulated genes were *BCL10* and *INPP5D*, respectively. Furthermore, we also identified significant lower expression of *CD79A* and *CD79B* (also known as Igα and Igβ, respectively). In a meta-analysis of GWAS results, Julià et al. reported BCR signaling pathway as the most significant biological process and *BCL10* and *CD79A* among top single markers associated with SLE [57].

Interestingly, our results addressed the dysregulation of several noteworthy node and edge parameters shared by both TCR and BCR signaling pathways. Among them *PI3K*/*AKT* signaling pathway and *VAV* gene homologs were altered; *AKT1*, *AKT2* and *VAV1*, *VAV2* expression levels were particularly reduced (Figs. 4 and 5). Previously, some researcher confirmed these results and proved phosphorylation level of *AKT* in SLE patients, which further support the major role of PI3K/Akt/TSC/mTOR signaling pathway in the disease [58, 59]. According to our results, the MAPK signaling pathway as an effective subnetwork of TCR and BCR signaling in SLE pathogenesis [59] also displayed a significant dysregulation, especially higher expression of *MAPK1*, *MAPK3*, and *PPP3CA* genes. Similarly, the strong association of *PPP3CA* with SLE-like disease has been described as new [60]. Moreover, we observed the reduced expression of JUN and FOS together with some increased or decreased biological relations with their neighbors in both TCR and BCR pathways. JUN and FOS are subunits of the transcription factor AP-1 which regulates cytokines and chemokines of TCR and BCR pathways in inflammatory and autoimmune disease [61].

Further investigation will be required to evaluate the efficiency of *CD80* and *CD86* → *CD28*, *C1R* and *C1QA* → *C2*, and *C1S* → *C4B* biological functions, PI3K/AKT signaling pathways, besides *JUN* gene as therapeutic targets in SLE disease. Moreover, analyzing the RNA-seq datasets could be better for distinguishing expression levels and biological relationships of genes’ isoforms in considered signaling pathways. It is necessary to examine the BNrich among some other autoimmune diseases in order to identify SLE signature genes more precisely.

## Conclusions

In the present study, we exploited BNrich to hybridize intergenic relations inferred from signaling pathways and gene expression profiling to improve the insight about SLE as a complex disease. We figured out the significant genes and intergenic relationships and systemic alterations in SLE, TCR, and BCR signaling pathways. Through cross-platform normalization and meta-analysis, large-enough SLE patients were analyzed. Since multiple platforms were utilized, the effects of individual study-specific biases were reduced.

Notably, the proposed computational methodology can be applied to a variety of complex diseases to illuminate new mechanistic insights in their pathogenesis.

## Supplementary information

**Additional file 1.** Batch effect removal results. The box plots and PCA plots related to CPN results for Hitachisoft and Illumina platforms.

**Additional file 2.** Significant and final parameters. The significant parameters of different platforms and the final parameters based on the mixture model in three examined signaling pathways.

## Data Availability

The R package of BNrich is available on Cran (https://cran.r-project.org/web/packages/BNrich/index.html). All gene expression data are available in the GEO database, and the raw data of signaling pathways are available in KEGG.

## Abbreviations

BNs: Bayesian networks
BCR: B cell receptor
CPN: Cross-platform normalization
CIA: Collagen-induced arthritis
FDR: False discovery rate
GEO: Gene Expression Omnibus
GSEA: Gene set enrichment analysis
GWAS: Genome-wide association studies
HC: Healthy control
IFN: Interferon
PEA: Pathway enrichment analysis
PBMC: Peripheral blood mononuclear cell
PCA: Principal component analysis
SLE: Systemic lupus erythematosus
Tfh: T follicular helper
TCR: T cell receptor
