# Supplementary material for: An integrative Bayesian network approach to highlight key drivers in systemic lupus erythematosus
Source: Arthritis Res Ther. 2020 Jun 23;22:156. doi: 10.1186/s13075-020-02239-3 (PMC7310461; doi:10.1186/s13075-020-02239-3)
Supplement: Supplementary file 1 — Additional file 1. Batch effect removal results. The box plots and PCA plots related to CPN results for Hitachisoft and Illumina platforms. [file 13075_2020_2239_MOESM1_ESM.docx]

Here we show the rest of the plots related to the cross platform normalization results.


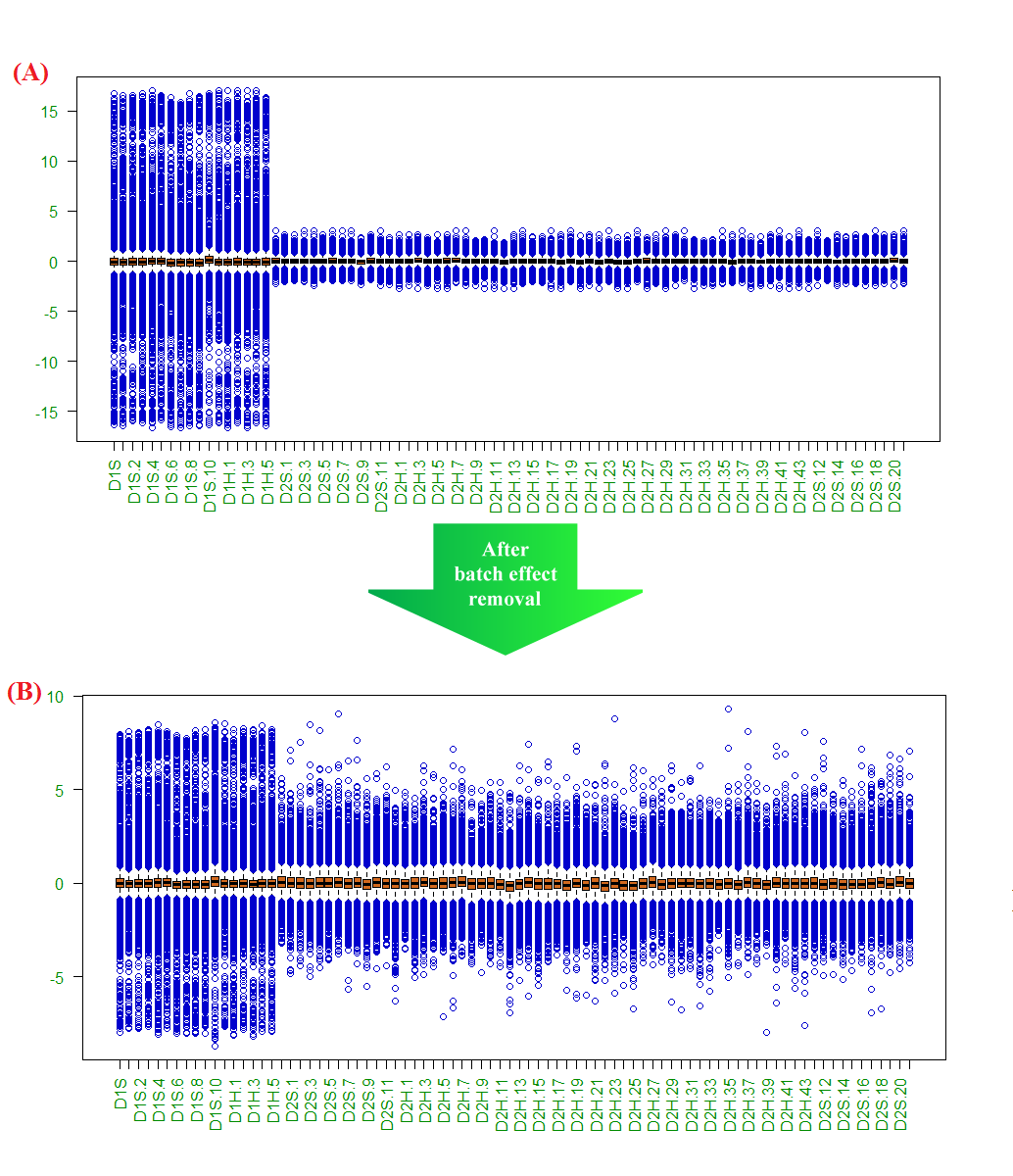


Figure A1. Graphical demonstration of the batch effect removal using ComBat. Boxplots for the gene expression distributions in Hitachisoft (A) before and (B) after batch removal. The distributions show the normalization and decreasing technical diversities between datasets. In the horizontal axis, the jth healthy control subjects and the jth SLE patient in ith dataset were illustrated with DiH.j and DiS.j, correspondingly.


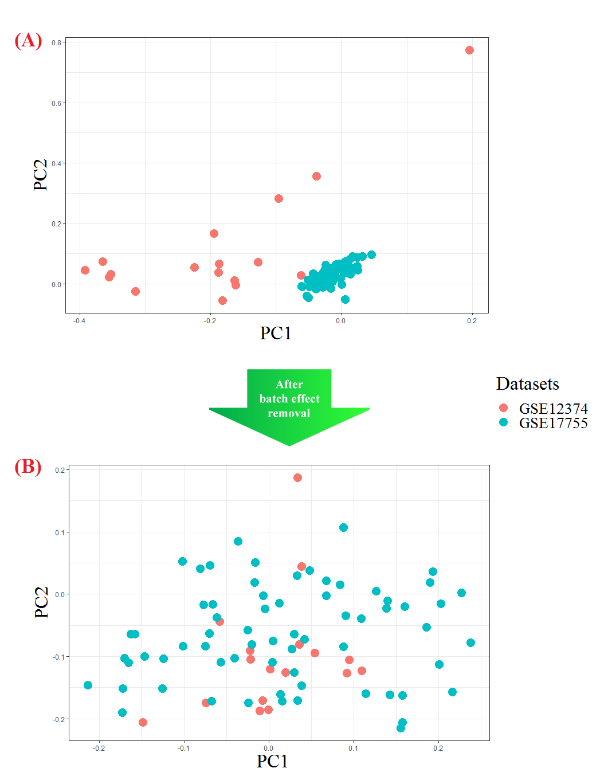


**Figure A2**. Graphical demonstration of the batch effect removal using ComBat. The PCA plots of the microarray datasets (A) before, and (B) after batch removal for Hitachisoft platforms. Each dot represents one sample and the color indicates its dataset.

**
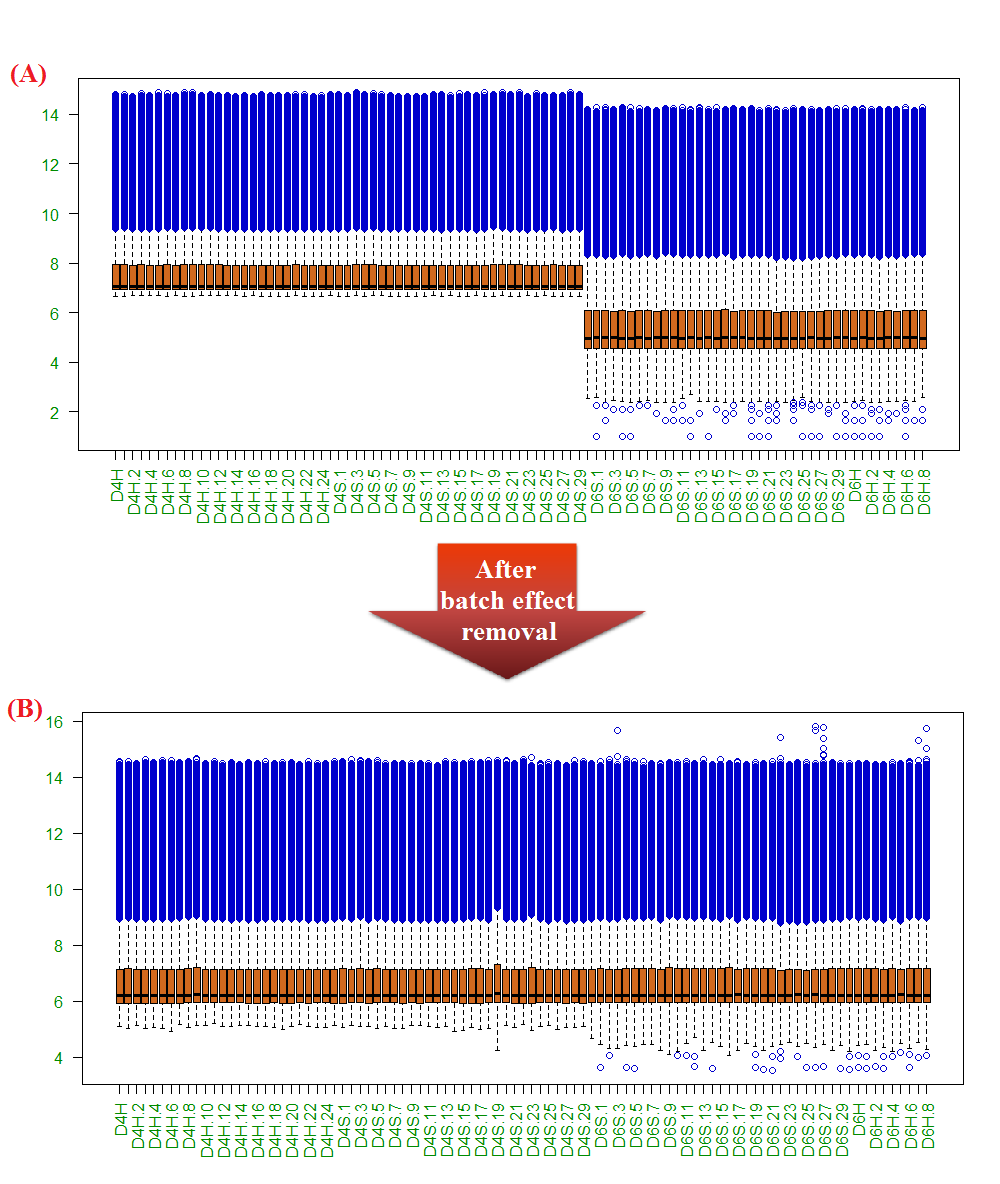
**

**Figure A3.** Graphical demonstration of the batch effect removal using ComBat. Boxplots for the gene expression distributions in Illumina (A) before and (B) after batch removal. The distributions show the normalization and decreasing technical diversities between datasets. In the horizontal axis, the jth healthy control subjects and the jth SLE patient in ith dataset were illustrated with DiH.j and DiS.j, correspondingly.


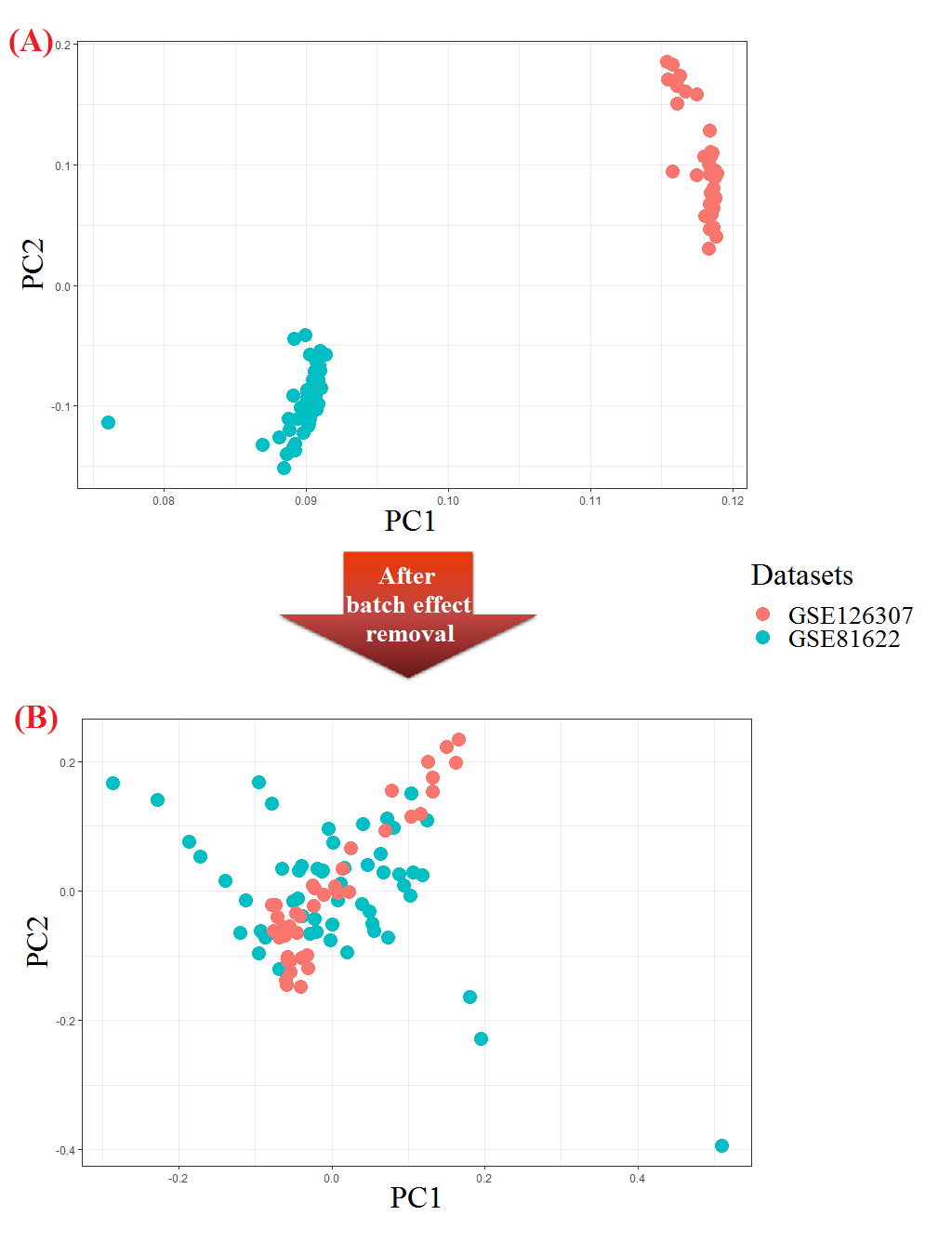


**Figure A2**. Graphical demonstration of the batch effect removal using ComBat. The PCA plots of the microarray datasets (A) before, and (B) after batch removal for Illumina platforms. Each dot represents one sample and the color indicates its dataset.
